# A Comparative In Vitro Analysis of Antimicrobial Effectiveness and Compressive Resilience in Chirata and Terminalia arjuna Modified Glass Ionomer Cement

**DOI:** 10.7759/cureus.52198

**Published:** 2024-01-13

**Authors:** Kamala Devi, Jessy Paulraj, Rinki S George, Rajeshkumar Shanmugam, Subhabrata Maiti

**Affiliations:** 1 Department of Pedodontics and Preventive Dentistry, Saveetha Dental College and Hospitals, Saveetha Institute of Medical and Technical Sciences, Saveetha University, Chennai, IND; 2 Department of Pharmacology, Saveetha Dental College and Hospitals, Saveetha Institute of Medical and Technical Sciences, Saveetha University, Chennai, IND; 3 Department of Prosthodontics, Saveetha Dental College and Hospitals, Saveetha Institute of Medical and Technical Sciences, Saveetha University, Chennai, IND

**Keywords:** terminalia arjuna modified gic, restorative dentistry, swertia chirata modified gic, compressive strength, antimicrobial, recurrent caries

## Abstract

Background: Glass ionomer cements are commonly utilized in dental restorations due to their biocompatibility, strong chemical bond with dental tissues, and ability to resist tooth decay. However, their effectiveness can be compromised by the presence of persistent cavity-causing microorganisms. Therefore, it is essential to consider incorporating antibacterial agents into these restorative materials. Swertia chirayita (S. chirayita) and Terminalia arjuna (T. arjuna) are well-known for their rich composition of phytochemicals that can potentially inhibit the growth of bacteria. Hence, the current research is focused on modifying glass ionomer cement with Chirayita and T. arjuna extracts to enhance its antibacterial properties.

Aim: This research aims to determine the antimicrobial efficacy and compressive strength of glass ionomer cement modified with Chirayita and T. arjuna extracts.

Methodology: Plant extracts were prepared from both Chirayita and T. arjuna. The powder and liquid components of conventional glass ionomer cement (GIC) were mixed, followed by adding these extracts at three different concentrations. To assess antimicrobial properties, typical strains of Streptococcus mutans and Lactobacillus were employed to test both the modified GIC and unmodified GIC (used as a control). For Chirayita and T. arjuna-modified GIC, minimum inhibitory concentration (MIC) assays were conducted at three different concentrations. MIC was assessed at various time intervals ranging from 1 to 4 hours for modified and unmodified groups. Moreover, compressive strength was measured using cylindrical molds. The highest force exerted at the point of specimen fracture was recorded to calculate the compressive strength values in megapascal (MPa).

Results: The antimicrobial efficiency of Chirata and T. arjuna-modified GIC was evaluated using a MIC assay, indicating a statistically significant enhancement in antimicrobial potency against S. mutans and Lactobacillus within the modified groups in contrast to the control group (p<0.05). However, there were no notable changes in compressive strength when comparing the control group to the modified groups (p>0.05).

Conclusion: The antimicrobial effectiveness against S. mutans was observed to be greater in both T. arjuna and Chirayita-modified GIC. In the case of Lactobacillus, Chirayita-modified GIC exhibited more pronounced antimicrobial properties compared to T. arjuna. Importantly, both extracts did not alter the compressive strength of Conventional (unmodified) GIC. Hence, Chirayita-modified GIC appears to be a promising restorative material for combatting recurrent caries. Additional investigation is required to assess the material's stability over an extended period.

## Introduction

Due to its unique properties, glass ionomer cement (GIC) is a commonly used material for restoring various dental lesions. However, its fluoride-releasing property alone is not sufficiently potent to prevent microbial growth and secondary caries [1,2]. Modifications have been implemented to improve the antimicrobial properties of conventional GIC. Several in vitro studies have shown that combining GIC with chlorhexidine (CHX) can enhance biological characteristics. However, after the addition, the material's mechanical properties are still questionable [3].

The Swertia genus, a member of the Gentianaceae family, encompasses around 135 species known for their medicinal properties. Among these Swertia chirayita (S. chirayita), a medicinal plant commonly referred to as chirayata or chirata is recognized for its diverse therapeutic capabilities [4]. Additionally, it possesses medicinal attributes such as antimicrobial, antimalarial, anti-amoebic, hypotensive, and antipsychotic effects. Chirayita contains various phytochemical compounds which contribute to its remarkable medicinal properties. Notably, antimicrobial screening of S. chirata indicates that these phytochemicals are most abundant in the leaves, stems, and root parts, suggesting their effectiveness against pathogenic microorganisms [5].

Terminalia arjuna (T. arjuna), commonly known as arjuna and belonging to the Combretaceae family, is a tree found in various regions of India and other countries, such as Burma, Sri Lanka, and Mauritius. Arjuna leaves are simple, oblong, or elliptic in shape [6]. This plant is also known for its ability to promote wound healing and the mineralization of bones [7]. Plants can produce secondary metabolites with antimicrobial properties, including tannins, terpenoids, alkaloids, flavonoids, glycosides, and phenols. Its leaves and bark are rich in glycosides with cardioprotective effects, flavonoids with anti-inflammatory and antibacterial properties, and tannins are known for astringent, antiviral, and antimicrobial activity [8].

The current scientific literature lacks sufficient support for incorporating T. arjuna extract and Chirayita into Glass Ionomer Cement for restorative purposes. Due to the rising incidence of recurrent cavities following restorative treatments, there is a need for a restorative material capable of inhibiting a wide range of microorganisms [9]. Existing literature has sought to establish this through reputable publications, yet there is a dearth of information in the realm of restorative dentistry towards these plant extracts [10-12]. As a result, our study aimed to assess the antibacterial properties of GICs modified with T. arjuna and Chirayita leaf extracts. Thus, with this objective in mind, this research was planned to evaluate and compare the antimicrobial effectiveness and compressive strength of modified GIC against conventional GIC. We hypothesized that there would be no significant difference between the two.

## Materials and methods

Study design and ethical approval 

An in-vitro study was conducted at the University Dental Hospital in India. Ethical clearance was obtained from the institutional review board (Ethical clearance number: SRB/SDC/UG-1817/22/PEDO/092).

Preparation of extract

**Figure 1:**
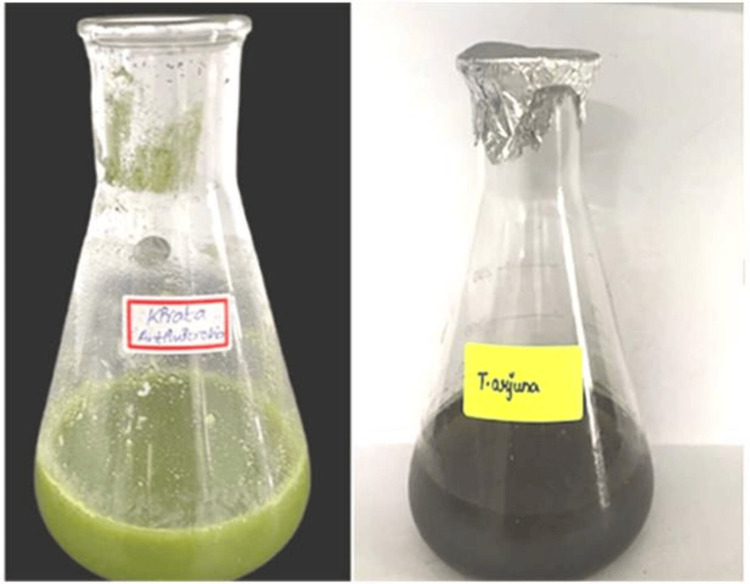
Chirata and T. arjuna leaf extract

S. chirayita and T. arjuna leaves were individually dried over five days. Glassware used in the process was thoroughly cleaned, washed, and then dried in a hot air oven before being utilized. To prepare two separate extracts, 1g of the respective leaves was measured and combined with 100 mL of distilled water in a beaker. The mixture was heated using a heating mantle at a temperature ranging from 60 to 70^o^C for 15 minutes. Subsequently, the solution was filtered, and the resulting filtrate was collected (Figure 1). This filtered extract was furthermore concentrated to a volume of 5 mL, maintaining a temperature between 60-70^o^C.

Inoculum preparation

The bacterial strains Streptococcus mutans and Lactobacillus acidophilus were cultured on Mueller Hinton Agar (MHA) to facilitate their growth. The microorganisms were subsequently subcultured in suitable culture media and individually inoculated into tubes containing 5 mL of sterile broth. These tubes were then incubated at a temperature of 37^o^C for 24 hours. Following incubation, the suspension was adjusted to a 0.5 McFarland scale.

Sample preparation

Type IX Glass Ionomer Cement (GIC) from GC Corporation was employed in this study. The different groups and their concentrations were tabulated (Table 1).

**Table 1:** Grouping

Groups	Description (P- Powder, Ex- Extract, SC- Swetia chirata, TA-Terminalia Arjuna, L- Liquid, GIC- Glass ionomer cement)
I	P^GIC^:SC^Ex ^: L^GIC^ = 2:1:1
II	P^GIC^: SC^Ex ^: L^GIC^ = 3:1:2
III	P^GIC^: SC^Ex ^: L^GIC^ = 3:2:1
IV	P^GIC^:TA^Ex ^: L^GIC^ = 2:1:1
V	P^GIC^: TA^Ex ^: L^GIC^ = 3:1:2
VI	P^GIC^: TA^Ex ^: L^GIC^ = 3:2:1
Control group	Conventional GIC(unmodified)

The plant extract was incorporated into conventional GIC by adding it to various combined liquid and powder component concentrations. Subsequently, the prepared specimens were swiftly placed in cylindrical wells using a sterile cement carrier, and the upper surface of the cement layer was leveled with a sterile glass slide within a minute. The finalized cement was poured into cylindrical molds, resulting in disc-shaped specimens with a thickness of 2 mm and a diameter of 6 mm after setting. The dimensions of each specimen were accurately measured with calipers. Each group comprised twelve specimens, including both S. mutans and Lactobacillus testing. Compressive strength assessment followed ISO 9917-1:2007 guidelines, utilizing cylindrical molds measuring 4.0 × 6.0 mm for each of the twelve specimens in every group. The molds were filled and leveled, and after removal from the mold an hour later, the samples were immersed in deionized water for 24 hours before evaluating compressive strength.

Minimal inhibitory concentration assay (MIC)

The sterile MHA broth, 200 µL in volume, was added to all seven wells. S. mutans and Lactobacillus bacterial strains of approximately 50 µL each, with a concentration of 5×105 CFU/ml, were added to all wells. The initial six wells held three distinct concentrations of modified GIC, with the final well serving as the control (Conventional GIC). Incubation was conducted under suitable conditions for different time intervals, ranging from the first to the fourth hour. The percentage of dead cells was determined at regular intervals by measuring the absorbance at 540nm using an ELISA reader.

Compressive strength evaluation

Defective specimens or those containing imperfections were eliminated from consideration. Using a digital micrometer gauge, the diameter of each specimen was verified. These specimens were oriented vertically within a Zwick universal testing machine (Instron, Electro Plus®, E3000). The compressive force was systematically exerted along the specimens' long axis at a rate of 0.5 mm/min until they fractured. The maximum force registered at the moment of fracture was documented to determine the compressive strength values expressed in MPa.

Data analysis

The collected data was input into a Microsoft Excel spreadsheet. Statistical analysis was conducted using SPSS version 24.0, involving descriptive analysis and repeated measure ANOVA for mean MIC values. To evaluate compressive strength, a one-way analysis of variance (ANOVA) was used for comparing the groups, with a significance level of p≤ 0.05.

## Results

Antimicrobial activity

**Figure 2:**
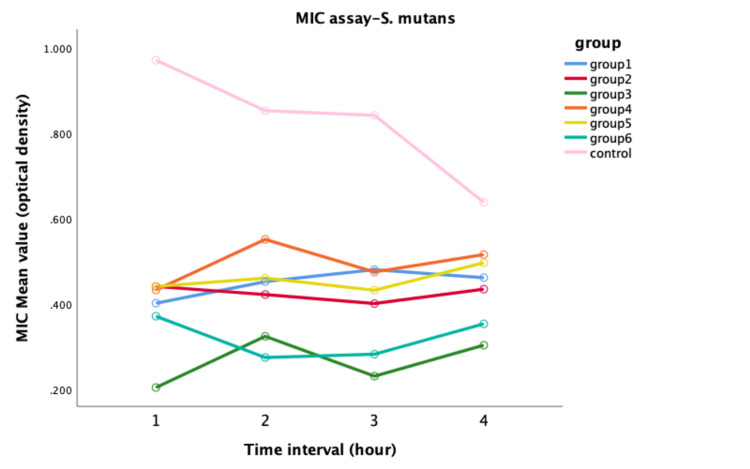
Antimicrobial efficacy against S. mutans between all groups

In the case of Streptococcus mutans (S. mutans), the modified groups showed superior performance than the control, with significant differences. A concentration ratio 3:2:1 for both extracts exhibited strong antimicrobial activity, which was determined through MIC assay (Figure 2).

Furthermore, the Tukey HSD test revealed a notable distinction between the control and remaining groups (p<0.05), as depicted in Table 2.

**Table 2:** Pairwise comparison of antimicrobial efficacy of Streptococcus mutans against a control group The p-value was significant at 0.05; the p-value was derived from the multiple comparisons of the Tukey HSD Test. HSD: honestly significant difference

Pair-wise comparison	Mean difference	95% Confidence interval		p-value
		Lower	Upper	
Group I vs. control	0.377	0.375	0.378	0.001*
Group II vs. control	0.401	0.400	0.402	0.001*
Group III vs. control	0.560	0.559	0.561	0.001*
Group IV vs. control	0.332	0.331	0.333	0.001*
Group V vs. control	0.368	0.367	0.369	0.001*
Group VI vs. control	0.505	0.504	0.506	0.001*

**Figure 3:**
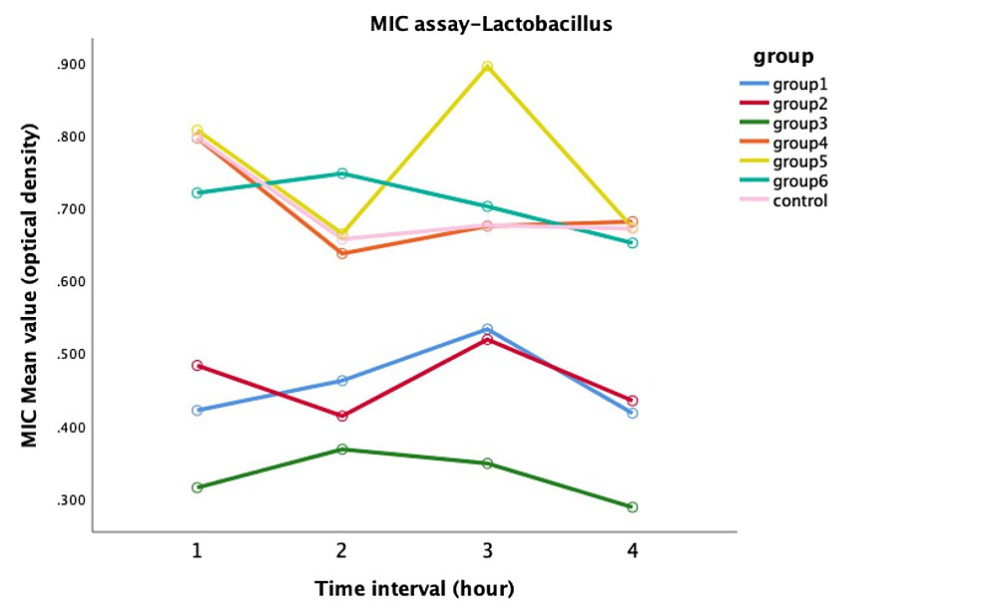
Antimicrobial efficacy against Lactobacillus between all groups

Significant variations in antimicrobial activity against Lactobacillus were observed between the modified and control groups. The repeated measure ANOVA linear chart showed against Lactobacillus Chirata-modified GIC displayed higher antibacterial activity than the T. arjuna groups (Figure 3).

In the pairwise comparisons, a statistically significant difference was evident when comparing the control group with the other groups (p<0.05) (Table 3).

**Table 3:** Pairwise comparison of antimicrobial efficacy on Lactobacillus against control groups The p-value was significant at 0.05; the p-value was derived from multiple comparisons of the Tukey HSD Test HSD: honestly significant difference

Pair-wise comparison	Mean difference	95% Confidence interval		p-value
		Lower	Upper	
Group I vs. control	0.242	0.241	0.243	0.001*
Group II vs. control	0.238	0.237	0.239	0.001*
Group III vs. control	0.371	0.370	0.372	0.001*
Group IV vs. control	0.003	0.002	0.004	0.001*
Group V vs. control	0.059	0.060	0.058	0.001*
Group VI vs. control	0.005	0.006	0.004	0.001*

Compressive strength

**Figure 4:**
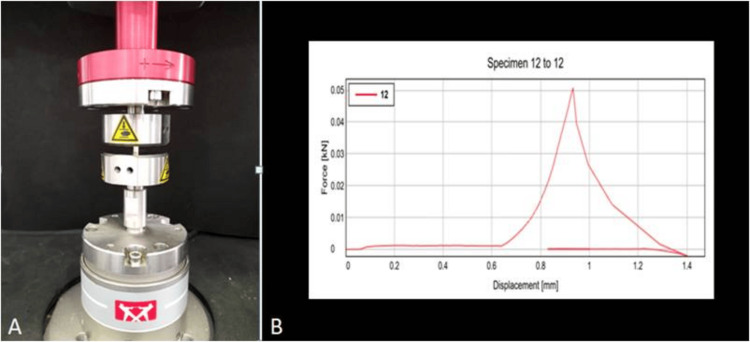
Compressive strength A-Testing, B- Linear graph of compressive strength of modified GIC GIC: glass ionomer cement

Compressive forces were exerted on the specimens, and the corresponding linear graph values were recorded (Figure 4).

To examine the variations in compressive strength among the groups, a one-way analysis of variance (ANOVA) was utilized. The results from the analysis showed no significant difference among the groups, as indicated by an F value of 0.810 and a p-value of 0.565 (Table 4). This implies that the control group performed similarly to the other groups.

**Table 4:** Comparison between groups for evaluation of compressive strength p-value was derived by one way ANOVA

Group	n	Mean ± SD	95 % Confidence interval		p-value
			Lower	Upper	
Group 1	12	174.4±1.80	173.3	175.6	0.565
Group 2	12	172.9±2.6	171.3	174.4	
Group 3	12	173.15±3.09	171.1	175.1	
Group 4	12	173.29±2.50	171.6	174.8	
Group 5	12	172.63±2.14	171.2	173.9	
Group 6	12	173.70±2.11	172.3	175.0	
Control	12	173.65±2.04	172.3	174.9	

## Discussion

The clinical effectiveness of dental restorative materials largely hinges on their ability to withstand the mechanical, chemical, and thermal stresses within the oral environment. Resistance to acidic challenges is crucial in ensuring the long-term durability of materials used in direct restorations. It has been shown that acidic conditions can have detrimental effects on tooth structure and restorative materials [13]. While adding antimicrobial agents to dental materials provides several therapeutic benefits, it frequently affects their physio-mechanical characteristics. These limitations underscore the need for innovative approaches to combat tooth decay without altering the material's inherent characteristics [14].

S. chirayita, a medicinal plant, is renowned for its ability to combat various bacterial infections. Chirayita contains a range of phytochemical compounds, including swertinin, swertianin, swerchirin, eniflavine, arginine, leucine, aspartic acid, and glutamic acid, all of which contribute to its impressive medicinal properties. Terminalia arjuna, another medicinal plant, boasts a diverse phytochemical profile featuring glycosides, flavonoids, tannins, triterpenoids, phenolics, and minerals. Flavonoids, well-known phytochemicals, are valued for their antimutagenic and antibacterial properties. Tannins, found in the bark of T. arjuna, are believed to possess antimicrobial potential, with constituents like pyrocatechol, castalagin, punicallin, punicalagin, and terflavin [15]. Interestingly, there is a paucity of prior research on the antimicrobial effects of GIC modified with T. arjuna and Chirayita against S. mutans and Lactobacillus. Therefore, our study was conducted to investigate the antimicrobial properties of these extracts.

Our current study observed significant antimicrobial activity against S. mutans, but the extracts demonstrated a more limited spectrum of antimicrobial activity against Lactobacillus. This limitation may be attributed to several factors. Firstly, the active compound in the crude extract may not be present in sufficient amounts to demonstrate activity at the used dosage, and secondly, if the active principle is abundant, other constituents in the extract could counteract the effects of the bioactive compound. [16]. Numerous prior in vitro studies have demonstrated the antibacterial properties of S. chirayita against various microorganisms [17,18]. Priyanka Roy et al. reported antimicrobial activity in the methanolic extract of both Swertia species stems and leaves [19]. Likewise, studies by Nyein L et al. highlighted the appreciable antibacterial properties of the ethanolic extract of S. chirayita in stems and leaves [20]. Jesmin Sultana et al. found that S. chirayita exhibited significant antibacterial and biological activities [21]. Syed RN et al. emphasized the antibacterial potential of S. chirayita, showing inhibition of Streptococcus growth, suggesting its potential for combating infectious and chronic diseases [22]. Furthermore, Arup Kumar Saha et al. conducted a study on dental caries prevention, confirming that S. chirayita displayed strong antimicrobial activity against S. mutans, similar to our current study, where it was incorporated into a restorative material [23]. Swertia chirayita's antibacterial properties are primarily linked to the presence of its biologically active components, including amarogentin, swerchirin, triterpenoids, xanthones, opelic acid, and gentiopicrin [17]. Additionally, key chemical compounds found in S. chirayita responsible for its antimicrobial properties include Swertiamarin, Sweroside, Swertanone, Oleanolic acid, and β-Amyrin [24].

Terminalia arjuna (TA) extracts in various solvent systems have demonstrated antimicrobial activity against diverse pathogens. The antimicrobial efficacy of this plant extends to periodontal pathogens, indicating its potential in treating periodontal diseases [25]. Interestingly, young branches of Terminalia arjuna are used as toothbrushes to alleviate toothaches. An in vivo study by Atul A Phatak et al. indicated that a poly-herbal extract mouthwash containing T. arjuna exhibited antimicrobial activity [26]. Mahima et al. (2013) emphasized the anti-cariogenic activity of T. arjuna bark and leaf extracts, suggesting that they interfere with the defense mechanisms of S. mutans, thereby controlling its growth and reducing the incidence of dental caries, which could be attributed to the antimicrobial activity of flavonoids and tannins [27]. Additional research by Shaswata Karmakar et al. proposed that Arjuna extract may serve as a valuable alternative therapeutic approach for addressing oral diseases associated with biofilms, such as periodontitis [25]. The above findings align with our study, highlighting the broad-range antibacterial activity of the extracts used. Active constituents like tannins, arjunic acid, arjunogenin, arjunetine, and arjunolone, identified in the bark, have been found to exhibit antimicrobial effects [7]. The presence of these compounds likely contributed to the results observed in our study. Nonetheless, the exploration of T. arjuna's antimicrobial activity remains limited, and its interaction with restorative materials has not been studied extensively, underscoring the significance of our present research.

Compressive strength is a critical factor to consider, given that most chewing forces exert compressive stress on dental restorations based on the location and the type of type of restoration. Therefore, we tested the compressive strength of this modified material. The results demonstrated that there was no significant change in compressive strength when compared to unmodified GIC. This indicates that the extract does not adversely affect the material's strength, aligning with a study by Farret et al. [28], which also found that adding antibacterial agents at precise concentrations did not influence the compressive strength properties of GIC. Our current findings support the idea that the modified material can serve as a viable alternative to conventional GIC for preventing recurrent caries, as it does not compromise material resilience. Therefore, Chirayita-containing GIC and T. arjuna-modified GIC may have clinical benefits in preventing secondary caries. By incorporating this bacteriostatic agent into GIC, it may be possible to prevent caries and restoration failures by inhibiting the growth of S. mutans. Hence, it can be applied clinically to individuals with deep dentinal caries, early childhood caries, rampant caries, and high caries indices. Thus, the combination of innovative materials, smart technologies, and individualized approaches has the capacity to revolutionize restorative dentistry, enhancing its effectiveness, longevity, and patient safety. Unfortunately, it's crucial to acknowledge that this study did not factor in intraoral variables such as typical masticatory forces, moisture levels, and variations in operator techniques. Therefore, additional investigations are required to evaluate the material's long-term durability.

## Conclusions

The findings demonstrated that Chirayita and T. arjuna-modified GIC performed better than the control group. Specifically, Chirayita-modified GIC exhibited more prominent antimicrobial properties compared to T. arjuna. Importantly, neither of the extracts adversely impacted the compressive strength of conventional GIC. Consequently, Chirayita modification can potentially enhance the dental applications of GIC as a restorative material for combating recurrent caries. However, additional studies are required to assess the material's durability over the long term and its behavior in vivo.
